# Case of Resection of the Right Elbow Joint for the Relief of Caries and Anchylosis of the Joint; Cured, with Considerable Motion at the Elbow

**Published:** 1855-02

**Authors:** Henry H. Smith

**Affiliations:** Consulting Surgeon to the Philadelphia Hospital, Blockley


### MEDICAL EXAMINER.

NEW SERIES.—NO. CXXII.—FEBRUARY, 1855.

### ORIGINAL COMMUNICATIONS.

*Case of Resection of the Right Elbow Joint for the relief of Caries and Anchylosis of the Joint; cured, with considerable motion at the Elbow.* By HENRY H. SMITH, M. D., Consulting Surgeon to the Philadelphia Hospital, Blockley.

Terence ———, aged 16 years, entered the Philadelphia Hospital, Feb. 23d, 1854, in consequence of caries of the right elbow and anchylosis of the joint, consequent on a fall.

The disorder having existed for 18 months, and the arm been allowed to become anchylosed in the straight position, he was sent as a pauper to the Alms House and thence to the Hospital. After being in the house about eight months, he was presented to me at the commencement of my term of service in October. At this time, he was pale, anæmic and enfeebled by long continued disease and suffering, and exhibited symptoms of hectic fever; the elbow-joint was very much swollen, and the skin inflamed and thickened round the bend of the elbow, several ulcerated spots and fistulous orifices existing both on the front and back of the arm. On introducing a probe into two or three of these orifices, the bones were readily felt in a carious condition; there was also perfect inability to move the hand in pronation or supination, the least attempt at bending the elbow or pronating the hand causing him severe pain. As his right arm was thus rendered useless, and there was every prospect of his either dying of hectic fever or becoming a pauper for life, I decided on attempting to save the limb by resecting the diseased joint. Accordingly, on the 11th of Oct. 1854, after an appropriate preliminary treatment, I operated as follows, before the medical class in attendance on the practice of the house.

*Operation.* The boy being fully etherized by a mixture of chloroform 1 part, ether 3 parts by weight, was laid on his belly with his face inclined to the side of the table, and a stout, round pillow placed on the front of the elbow as a support to the portion to be operated on, as well as with the view of favoring the flexion of the fore-arm, after the section of the olecranon process.

The arm being thus steadied, an H incision was made over the joint on its posterior face; the flaps turned up, the ulna nerve dissected out of its trochlea, and held on the front side of the internal condyle, and the artery which accompanied it compressed at a point where it was wounded. Martin's* circular saw being then applied to the shaft of the ulna, the olecranon process was soon removed, and the whole joint being thus laid open and found to be diseased, both the condyles of the humerus, as well as the epitrochlea, were sawed off by the same saw. The head of the radius being also diseased, it was excised from the neck of the bone by means of large and strong bone-nippers. So little hemorrhage ensued on the operation that no ligatures were applied. The flaps were then loosely united by sutures, supported by a light bandage, and the boy placed in bed with the arm supported on a pillow in the semi-flexed position, the whole elbow being covered by cloths, wrung out of tepid water. At 8 o'clock, P. M., his pulse was 90, and, as he was suffering, 60 drops of laudanum were given to allay the pain.

*Oct. 12th.* Slept well, better than for many weeks; suffers but little. Ordered chicken-broth and anodyne pro re nata.

*Oct. 14th.* Removed dressing; suppuration commenced; ordered a light bandage to the part; omit water dressing; tinct. cinchonæ compos. f℥iv. per diem; chicken for dinner.

[* This saw may be found figured in Smith's Operative Surgery, second edition, plate 5, fig. 1, vol. 1.—Editor.]

*Oct.* 16*th.* Dressed arm and increased the flexion slightly.

*Oct.* 18*th.* Applied an obtuse angular splint to the front of the arm; ordered to sit up.

*Oct.* 20*th.* Has his clothing on.

*Oct.* 24*th.* Applied a splint nearly of a right angle.

After this date, the wound was dressed daily, the angle of the splint being gradually changed to a right angle, and then to one which semi-flexed the arm.

*Dec.* 5*th.* Terence is now able to do without the splint, and has considerable motion at the joint, the wound being healed.

*Jan.* 15*th.* Terence can now move his elbow, so that his hand will traverse an arc of 40 degrees, and can pronate and supinate the hand quite well.

*Remarks.* The advantages of the operation of resection in this case are so apparent as not to require much argument, the saving of a right arm in one dependant on his daily labor for his support, being sufficient evidence of its value as a means of treatment in similar cases.

As a class, few operations are more strikingly illustrative of the progress of conservative surgery than those of resections, yet the number of instances reported in the United States of its application to the upper extremity are by no means commensurate with the cases which might have thus escaped amputation. After a careful examination of a very considerable number of American medical periodicals I find only the following:—one by Dr. Thos. Harris, of Philadelphia, in 1836; one by Dr. Gurdon Buck, Jr., of New York, in 1841, another in 1843, and a third one in 1846; and one by Dr. J. Pancoast in 1842. These cases, with the present one, making only six instances in which this operation has been published. In every case no serious symptoms supervened on the operation, and the patient was relieved of the exhaustion and suffering attendant on the disease, besides obtaining a comparatively useful limb.

The entire head of the humerus was resected by Dr. Hunt, of Washington city, in 1818, and a large portion of the same bone (its head) was removed by Dr. Pinckney, of the Navy, in 1846. When we compare this limited number of operations with the numerous cases of diseased joints that have required it, we must admit that resections of the bones of the upper extremity have not received the attention that the benefits conferred by the operation might naturally lead us to anticipate, and it is with a view of calling professional notice to this useful class of operations that the present case has now been reported.

